# Understanding Equity in Cancer Prehabilitation Services in Wales: A Medical Record Review

**DOI:** 10.1002/cnr2.70598

**Published:** 2026-06-11

**Authors:** Shea Palmer, Alexandra Mitchell, Akhilesh Ramachandran, Nichola Gale, Sian Lewis, Manasi Patil, Nicholas Courtier, Jane Hopkinson

**Affiliations:** ^1^ School of Healthcare Sciences Cardiff University Wales UK; ^2^ Aneurin Bevan University Health Board Wales UK

**Keywords:** cancer, ethnicity, health equity, prehabilitation, socioeconomic

## Abstract

**Background:**

Cancer prehabilitation prepares people for cancer treatment by promoting physical activity, eating well and emotional wellbeing. Evidence shows that prehabilitation before and during cancer treatment reduces treatment complications and improves outcomes, including survival. However, the availability of and patient engagement with prehabilitation is variable. People from lower socioeconomic and minority ethnic backgrounds are at greater risk of poor health outcomes from diseases like cancer and are less likely to engage with prehabilitation services. Access, acceptance and adherence to prehabilitation for all cancer patients is important for progress towards equity in treatment outcomes. Understanding how prehabilitation services are currently delivered is an important first step in improving cancer health equity. The medical record review reported here was conducted as part of case study research on equity of cancer prehabilitation services in Wales.

**Aims:**

To describe cancer prehabilitation services delivered across the National Health Service (NHS) in Wales, with a view to understanding issues related to health equity.

**Methods and Results:**

Data were collected from medical records for all patients who attended an initial cancer prehabilitation consultation over a four‐week period at each of seven NHS providers. Patients were included if they were adults referred for prehabilitation with a confirmed or suspected diagnosis of upper gastrointestinal, colorectal, lung, prostate or breast cancer and were awaiting active or palliative treatment. Data extracted for each patient included demographics, cancer site and the components of prehabilitation received. The Charlson Comorbidity Index and Index of Multiple Deprivation were calculated. Descriptive statistics and correlational analysis were used. A total of 134 individual patient cases were included. Of these, 63% had colorectal cancer, 22% upper gastrointestinal cancer and 12% lung cancer. Mean age was 68 years, with 56% men and 44% women. Forty‐two percent were from the two most deprived quintiles, whilst 100% of patients with a recorded ethnicity were White—neither figure is representative of cancer cases in Wales. There was wide variability across services in the type and duration of prehabilitation received. An estimated 14% of patients newly diagnosed with the included cancers in Wales accessed prehabilitation.

**Conclusion:**

The findings demonstrate a highly variable prehabilitation offer in Wales. Targeted efforts are required to improve uptake among socioeconomically disadvantaged groups to enhance cancer treatment outcomes.

## 1 Introduction

Prehabilitation prepares people for cancer treatment. It promotes proactive behaviours for recovery and prescribes exercise, nutrition and psychological interventions [1]. The existing research is in its infancy and has methodological limitations, including exploratory single cohort studies with small sample sizes and a high risk of bias [2, 3]. However, there is a growing body of evidence showing that cancer prehabilitation reduces morbidity and mortality [4, 5, 6]. It can lead to fewer treatment complications and improved outcomes that include longer and better quality life [7, 8].

However, the availability of and patient engagement with cancer prehabilitation is variable [9]. People from lower socioeconomic groups are known to be at greater risk of poor health outcomes compared to people from higher socioeconomic groups [10, 11, 12]. It is also known that some patients from socioeconomically disadvantaged backgrounds and some minority ethnic groups are less likely to take up prehabilitation services [13]. It is essential to enable access, acceptance and adherence to prehabilitation for all people with cancer, particularly as those groups are likely to gain greatest benefit.

There are different models of prehabilitation service delivery described in the literature, including unimodal, bimodal and multimodal approaches which might be delivered face to face or virtually [14]. A comprehensive international survey found variability in prehabilitation practices across the world for patients undergoing cancer surgery [15]. Although there is a growing literature supporting the benefits of prehabilitation, there is no consensus on the optimal service delivery. In order to maximise the benefits of prehabilitation, it is first important to understand who is accessing services and the nature of the interventions they are receiving.

In Wales, there was a 25% increase in new cancer diagnoses from 2002 (16 600 cases) to 2019 (20 058 cases) [16]. The general population is increasing and there is a rise in the proportion of older people who are more likely to receive a cancer diagnosis. In 2021, the four most diagnosed cancers in Wales were breast cancer (BC, 2952 cases), colorectal cancer (CRC, 2654 cases), prostate cancer (PC, 2518 cases) and lung cancer (LC, 2469 cases), together accounting for more than 50% of all cancer cases [17, 18]. The current retrospective medical record review therefore focused on prehabilitation provision for these four cancer sites. Upper gastrointestinal cancer (UGIC, 2105 cases [17, 18]) was also included because of the growing evidence base for benefit from prehabilitation in this group [19].

The aim of this medical record review was to describe cancer prehabilitation services delivered across the National Health Service (NHS) in Wales. Four research questions were addressed: (1) What proportion of patients awaiting cancer treatment access prehabilitation services? (2) What are the sociodemographic characteristics of patients who access prehabilitation? (3) What proportion of patients who access prehabilitation are in groups (ethnic and socioeconomic) at higher risk of poor treatment outcome? and (4) What is the duration and composition of prehabilitation received by those accessing services?

## 2 Methods

This retrospective medical record review was part of case study research exploring access to and engagement with cancer prehabilitation services in Wales, which in turn was part of the wider Inclusive Prehabilitation (‘I‐Prehab’) project aiming to improve the inclusivity of prehabilitation for people with cancer (https://fundingawards.nihr.ac.uk/award/NIHR151668). Qualitative observations, interviews and focus groups were also conducted as part of the case study research but will be reported separately. A systematic review exploring access, acceptance and adherence to cancer prehabilitation has already been published [20]. Ethical approval was granted by London‐Surrey Borders NHS Research Ethics Committee (Reference 23/PR/0868). Local research governance approvals were also secured from each participating service provider. As only anonymised data was extracted from medical records, individual patient consent was not required.

Each of the seven NHS Health Boards and one NHS Trust in Wales caring for patients with cancer were approached to take part. All eight NHS organisations reported that a cancer prehabilitation service was offered to patients in their care. However, it was established during study set‐up that one commissioned cancer services, including prehabilitation, from other NHS organisations in Wales and England. Seven of the eight providers in Wales were thus included in the medical record review.

The study team contacted each service with a request to collect data from medical and prehabilitation records over a 4‐week period, immediately prior to qualitative data collection within that service. The medical record review was conducted from September 2023 to August 2024. An administrator or clinician at each study site was trained by a researcher in the use of a data extraction tool (Supporting Information Material 1). Data extracted included information regarding patient demographics; cancer site; planned cancer treatment; comorbidities and details of prehabilitation interventions delivered (classified as support for exercise, nutrition and/or emotional wellbeing). Postcode was used to assign individuals to an Index of Multiple Deprivation (IMD) quintile, with 1 being the most deprived and 5 the least deprived [21]. The age‐adjusted Charlson Comorbidity Index (CCI) was calculated for individual cases based on recorded comorbidities. The CCI predicts mortality based on the number and severity of comorbidities adjusted for age [22]. Higher scores relate to higher predicted mortality and lower predicted 10‐year survival [23, 24].

### 2.1 Eligibility Criteria

Patients were included if they: were ≥ 18 years old; were referred for prehabilitation and received at least one session within the four‐week data collection period; had a confirmed cancer diagnosis (BC, CRC, PC, LC or UGIC) or high suspicion of cancer at the point of prehabilitation referral; were awaiting active treatment (with curative or palliative intent) for primary, locally advanced or metastatic cancer. Patients with high suspicion of cancer were included because they are placed on the same urgent referral pathway whilst they await diagnostic confirmation. For the purposes of this study, UGIC included head and neck, cholangiocarcinoma, gallbladder, liver, stomach and pancreatic cancer. Patients were excluded if the prehabilitation referral was for a condition other than cancer; they were receiving supportive care or active surveillance with no plan for treatment; they declined cancer prehabilitation or cancelled or did not attend the first prehabilitation assessment.

### 2.2 Data Analysis

Data were anonymised by the NHS administrator or clinician prior to transfer to the research team for entry into a central database (IBM SPSS Statistics, Version 23). Data entry, cleaning and coding were conducted by one researcher (A.R.) and checked and verified by two others (J.H. and A.M.). Processes included verifying eligibility; identifying and correcting missing data through liaison with services; verifying entered data against the original data extraction tools and allocating numerical codes to textual data, for example, to classify prehabilitation interventions. Codes were discussed and agreed by A.R., J.H. and A.M. Each of the seven service providers was randomly allocated a letter identifier (A–G) to preserve anonymity.

Descriptive statistics (numbers and proportions) were used to summarise the data. IMD quintiles were sometimes aggregated into most deprived (quintiles 1 plus 2) and least deprived (quintiles 4 plus 5) to provide larger case numbers and more meaningful comparisons. Exploratory Spearman's *ρ* correlation analysis was used to consider the relationships between relevant ordinal and scale variables (e.g., between IMD and CCI). Exploratory Kruskal–Wallis tests were also used to identify differences in relevant variables (e.g., the duration of prehabilitation across IMD quintiles). Such exploratory inferential analyses were only performed at a whole sample level due to the likelihood of Type II errors associated with sub‐group analysis. Where relevant, the results have been placed in context using other sources of publicly available data.

## 3 Results

### 3.1 Sample

Data were collected and submitted from 167 individual cancer cases across seven cancer service providers. A final sample of 134 cases met the eligibility criteria and were included in analysis (Table 1). Reasons for exclusion were a noncancer diagnosis; no cancer treatment planned; cancer treatment declined by patient; cancer treatment already started or a noneligible cancer diagnosis.

**TABLE 1:** Included cases and summary description of each service.

Provider	Cases (*n*)	Service description	Delivery mode	Patient group and enrolment criteria
A	22	Support worker‐led screening service (universal advice and onward referral)	Telephone	CRC
B	21	Multidisciplinary prehabilitation service	Online (virtual) or telephone	CRC, PC, oesophageal and gynaecological cancer; ≥ 2 weeks before surgery or treatment; referrals risk stratified: (1) specialist, (2) targeted, (3) universal
C	36	Multidisciplinary prehabilitation service	Face‐to‐face, some classes online (virtual)	CRC, LC, UGIC, LC, hepato‐pancreato‐billary, ovarian and urological, cancer (suspected or diagnosed); ≥ 2 weeks before surgery; referrals risk stratified: (1) specialist, (2) targeted, (3) universal
D	23	Multidisciplinary prehabilitation service	Face‐to‐face, some classes online (virtual)	CRC or UGIC; high point of suspicion for cancer diagnosis; Referrals risk stratified: (1) specialist, (2) targeted, (3) universal
E	9	Physiotherapy‐led prehabilitation service	Face‐to‐face	Suspected LC requiring surgery
F	8	Multidisciplinary prehabilitation service	Face‐to‐face	BC, CRC, LC, UGIC and gynaecological, cancer; assessed pre‐operatively as high risk for urgent major surgery
G	15	Consultant‐led clinics, with clinical nurse specialist and dietitian prehabilitation advice	Face‐to‐face	UGIC; chemotherapy ± radiotherapy
Total	134	—	—	—

Abbreviations: BC = breast cancer; CRC = colorectal cancer; LC = lung cancer; PC = prostate cancer; UGIC = upper gastrointestinal cancer.

### 3.2 Overview of Prehabilitation Services

The prehabilitation service received across the seven providers was diverse, as summarised below and in Table 1.

At Provider A, patients newly diagnosed with CRC were screened by a support worker, received universal verbal advice and were signposted to web‐based information. If appropriate, they were also referred to clinical experts such as a registered dietitian. Provider A therefore did not offer a comprehensive prehabilitation service to support exercise, nutrition and emotional wellbeing [1].

Three providers offered a multidisciplinary prehabilitation service dedicated to broader groups of cancer patients. Provider B offered an online, virtual multidisciplinary prehabilitation service. Providers C and D had face‐to‐face multidisciplinary prehabilitation teams and facilities, although some classes were delivered online. All three of these providers (B, C and D) offered all components of prehabilitation (exercise, nutrition and emotional wellbeing) tailored to individual patient need. Prehabilitation was available to those with a suspected cancer diagnosis and to those with a confirmed diagnosis awaiting treatment, although each service had nuanced referral criteria based on factors such as cancer site, planned treatment and time available before treatment.

Two providers offered prehabilitation to targeted cancer patient groups. Provider E offered a physiotherapy‐led service primarily for patients with lung cancer undergoing surgery, although some received other treatments or had other primary cancers with lung metastasis. Patients were offered face‐to‐face support for improving respiratory function and general fitness, based on local and research‐based evidence of benefit. Provider F offered a face‐to‐face multidisciplinary prehabilitation service but only to patients with a cancer diagnosis who were identified as being at high risk of poor outcome from surgery. Engagement with and improvements in response to prehabilitation might inform the decision to proceed with surgery.

Provider G, a specialist tertiary treatment centre, offered prehabilitation advice within consultant‐led clinics for patients with UGIC before and during treatment. Clinics included clinical nurse specialist and dietitian input and prehabilitation advice was incorporated into dietetic consultations. Some patients commenced prehabilitation at their referring health board and were transferred once a decision was made to implement treatment at the tertiary centre. Other patients continued to access prehabilitation across multiple services.

Results relevant to each of the research questions are detailed in the following sections:

### 3.3 Question 1: What Proportion of Patients Awaiting Cancer Treatment Access Prehabilitation Services?

This question necessitated comparison with publicly available data. In 2021, the combined annual incidence of BC, CRC, LC, PC and UGIC in Wales was 12 698 [17, 18], equating to approximately 974 every 4 weeks. This suggests that approximately 14% (134/974) of patients diagnosed with these cancers access services offering prehabilitation in Wales. Caution needs to be taken with this estimate due to the historical nature of the denominator data and because some patients may have received prehabilitation in England, although such numbers are likely to have been very small and unlikely to substantially alter the calculation.

### 3.4 Question 2: What Are the Sociodemographic Characteristics of Patients Who Access Prehabilitation?

#### 3.4.1 Ethnicity

Three service providers did not routinely collect information related to the ethnicity of people accessing cancer prehabilitation. Ethnicity was therefore only recorded in 87 cases (87/134, 65%), of which all (100%) were recorded as ‘White’ (either Welsh, English, British, Scottish or Northern Irish).

#### 3.4.2 IMD

From a sample of 134 patients, *n* = 24 (18%) were from Quintile 1 (most deprived), *n* = 32 (24%) Quintile 2, *n* = 27 (20%) quintile 3, *n* = 20 (15%) Quintile 4 and *n* = 31 (23%) Quintile 5 (least deprived). The median quintile was 3. Thus, 56/134 patients (42%) were from the most deprived two quintiles and 51/134 (38%) from the least deprived two quintiles.

#### 3.4.3 Cancer Site

The 134 cases comprised of people with CRC (*n* = 85), UGIC (*n* = 29), LC (*n* = 16), PC (*n* = 3) and BC (*n* = 1). At the time of accessing prehabilitation, 81% (109/134) had a confirmed cancer diagnosis and 19% (25/134) had a high suspicion of cancer.

#### 3.4.4 Age

Patients ranged from 36 to 89 years old (mean ± SD 68 ± 10 years). The relevant values for individual cancer sites were UGIC = 67 ± 9, CRC = 68 ± 10 and LC = 72 ± 7. It was not meaningful to calculate the mean age for PC and BC due to small numbers.

#### 3.4.5 Gender

The total sample was 56% male (75/134) and 44% female (59/134). UGIC and CRC had a higher proportion of males (66% and 58% respectively), whereas LC had a higher proportion of females (75%).

#### 3.4.6 Reason for Prehabilitation Referral

Of those accessing prehabilitation, 50% (67/134) were referred prior to surgery; 14% (19/134) prior to chemotherapy; 6% (8/134) prior to chemoradiotherapy; 2% prior to radiotherapy (3/134) and 25% (34/134) for pretreatment fitness or optimisation support. Treatment was still to be decided for 2% (3/134).

### 3.5 Question 3: What Proportion of Patients Who Access Prehabilitation Are in Groups (Ethnic and Socioeconomic) at Higher Risk of Poor Treatment Outcome?

Due to the lack of ethnic diversity in the sample, this analysis focused only on IMD data as a potential indicator of poor treatment outcome. IMD profiles were also explored by cancer site, service provider and CCI to explore other potentially relevant factors influencing prehabilitation uptake (Table 2).

**TABLE 2:** IMD by cancer site, service provider and Charlson Comorbidity Index.

	IMD quintile (1 = most deprived, 5 = least deprived)					Total (*n*)
	1	2	3	4	5	
Number of patients, *n* (%)						
Number	24 (18%)	32 (24%)	27 (20%)	20 (15%)	31 (23%)	134
Cancer site, *n* (%)						
CRC	12 (14%)	20 (24%)	14 (17%)	15 (18%)	24 (28%)	85
LC	4 (25%)	2 (13%)	5 (31%)	2 (13%)	3 (19%)	16
UGIC	7 (24%)	10 (34%)	6 (21%)	2 (7%)	4 (14%)	29
Provider, *n* (%)						
A	2 (9%)	5 (23%)	5 (23%)	1 (5%)	9 (41%)	22
B	2 (10%)	4 (19%)	5 (24%)	7 (33%)	3 (14%)	21
C	11 (31%)	6 (17%)	8 (22%)	3 (8%)	8 (22%)	36
D	4 (17%)	9 (39%)	4 (17%)	2 (9%)	4 (17%)	23
E	0 (0%)	2 (22%)	3 (33%)	2 (22%)	2 (22%)	9
F	1 (13%)	0 (0%)	1 (13%)	4 (50%)	2 (25%)	8
G	4 (27%)	6 (40%)	1 (7%)	1 (7%)	3 (20%)	15
Charlson Comorbidity Index (CCI), median (inter quartile range [IQR])						
CCI	5 (4)	6 (4)	6 (2)	5 (3)	6 (3)	N/A

Abbreviations: CRC = colorectal cancer; LC = lung cancer; UGIC = upper gastrointestinal cancer.

Overall, there was a slightly higher proportion of patients from the two most deprived quintiles (42%) compared to the two least deprived quintiles (38%).

Investigation of IMD by cancer site revealed a higher proportion of people from the most deprived areas for LC (38% in the most deprived two quintiles versus 32% in the least deprived two quintiles) and UGIC (54% vs. 21%), but not for CRC (38% vs. 46%). BC (*n* = 1) and PC (*n* = 3) were excluded from analysis due to very small numbers.

Investigation of IMD by provider reveals differences. There was a higher proportion of people from the most deprived areas for three providers (Provider C 48% from the two lowest quintiles vs. 30% from the highest two quintiles; Provider D 56% vs. 26% and Provider G 67% vs. 27%). However, the opposite was observed for the other service providers (Provider A 32% vs. 46%; Provider B 29% vs. 47%; Provider E 22% vs. 44% and Provider F 13% vs. 75%).

The age‐adjusted CCI scores showed little difference across IMD quintiles. There was no correlation between IMD and CCI values (Spearman's *ρ* −0.04, *p* = 0.68).

### 3.6 Question 4. What Is the Duration and Composition of Prehabilitation Received by Those Accessing Services?

As Provider A only offered a screening service, they were excluded from this element of the medical record review. Therefore, six providers of prehabilitation are described here (*n* = 112 cases).

#### 3.6.1 Duration of Prehabilitation

The time attending prehabilitation ranged from 1 to 127 days (median (IQR) 16.5 (30) days). People from IMD 3 spent longest in prehabilitation (median 28 days) and those from IMD 4 the least (median 9.5 days) (Table 3). However, there was no statistically significant difference across IMD quintiles (Kruskal–Wallis test, *p* = 0.19). The correlation between IMD and time spent in prehabilitation was very weak (Spearman's *r* = −0.08) and not statistically significant (*p* = 0.42). The correlation between age and time spent in prehabilitation was also very weak (*r* = 0.17) and nonsignificant (*p* = 0.09).

**TABLE 3:** Duration of prehabilitation by IMD.

	IMD quintile (1 = most deprived, 5 = least deprived)					Total, *n*
	1	2	3	4	5	
Duration of prehabilitation, median (IQR), days						
Time	14 (40)	21.5 (23)	28 (30)	9.5 (14)	14.5 (34)	16.5 (30)

Duration of prehabilitation was different for different cancer sites, being longer for people with LC compared to CRC and UGIC (median 55 vs. 26 days and 16 days, respectively). Again, BC and PC were excluded from analysis due to very low numbers. The difference across cancer sites for time spent in prehabilitation was statistically significant (Kruskal–Wallis, *p* < 0.01). Pairwise comparisons were not conducted due to small numbers.

Duration of prehabilitation also varied depending on the reason for prehabilitation referral (Table 4). Those referred for pretreatment fitness or optimisation support (encompassing responses recorded by clinical teams such as ‘optimise prior to treatment’ and ‘improve fitness levels’) received the longest prehabilitation, although it should be noted that many of those patients may have gone on to receive one of the other specified treatments. The difference across referral reasons was statistically significant (Kruskal–Wallis, *p* = 0.030) but pairwise comparisons were not undertaken, again because of small numbers in some subgroups.

**TABLE 4:** Duration of prehabilitation by reason for referral.

Reason for prehabilitation referral	Duration of prehabilitation, median (IQR), days
Surgery	16 (31)
Chemotherapy	4.5 (26)
Radiotherapy	1 (0)
Chemoradiotherapy	12 (12)
Treatment to be decided	17 (21.5)
Pretreatment fitness or optimisation support	28 (41)

The duration of prehabilitation differed between providers, with the highest median number of days recorded for Provider E (Table 5). The difference across all providers was statistically significant (Kruskal–Wallis *p* < 0.01) but further pairwise comparisons were not performed to avoid Type II errors.

**TABLE 5:** Duration of prehabilitation by service provider.

Provider	Duration of prehabilitation, median (IQR), days
B	8 (23)
C	25 (29)
D	21 (21)
E	78 (65)
F	13.5 (32)
G	4 (10)

#### 3.6.2 Components of Prehabilitation

For this study, components of prehabilitation were classified as being related to either (i) physical activity, exercise or functional capacity, including inspiratory muscle training (abbreviated to ‘exercise’); (ii) diet or nutrition (abbreviated to ‘nutrition’) or (iii) emotional wellbeing or psychological support (abbreviated to ‘emotion’). One or more interventions were delivered in 110/112 cases. In two cases, an assessment was conducted but no intervention was delivered.

The number of prehabilitation components received differed across service providers (Tables 6 and 7). Some sites did not deliver all three prehabilitation components. For example, Provider G was a tertiary treatment centre for people with UGIC that primarily offered support for nutrition, sometimes alongside emotional support, but it did not provide support for exercise. Whereas Provider F, which was a specialist service for people at high risk from cancer surgery, always delivered all three components.

**TABLE 6:** The number of prehabilitation components received by people at each service provider (components were defined as support for exercise, nutrition and emotion).

	No intervention	1 component	2 components	3 components	Total
Provider B	1 (5%)	0 (0%)	3 (14%)	17 (81%)	21
Provider C	0 (0%)	6 (17%)	21 (58%)	9 (25%)	36
Provider D	1 (4%)	5 (22%)	13 (57%)	4 (17%)	23
Provider E	0 (0%)	8 (89%)	1 (11%)	0 (0%)	9
Provider F	0 (0%)	0 (0%)	0 (0%)	8 (100%)	8
Provider G	0 (0%)	13 (%)	2 (%)	0 (0%)	15
Total	2 (2%)	32 (29%)	40 (36%)	38 (34%)	112

**TABLE 7:** Specific combination of prehabilitation components received by people at each service provider and overall (components were defined as support for exercise, nutrition and emotion).

	Exercise only	Nutrition only	Emotion only	Exercise + Nutrition	Exercise + Emotion	Nutrition + Emotion	Exercise + Nutrition + Emotion	No intervention (assessment only)	Total
Provider B	0 (0%)	0 (0%)	0 (0%)	0 (0%)	3 (14%)	0 (0%)	17 (81%)	1 (5%)	21
Provider C	3 (8%)	3 (8%)	0 (0%)	18 (50%)	2 (6%)	1 (3%)	9 (25%)	0 (0%)	36
Provider D	3 (13%)	2 (9%)	0 (0%)	9 (39%)	0 (0%)	4 (17%)	4 (17%)	1 (4%)	23
Provider E	8 (89%)	0 (0%)	0 (0%)	0 (0%)	1 (11%)	0 (0%)	0 (0%)	0 (0%)	9
Provider F	0 (0%)	0 (0%)	0 (0%)	0 (0%)	0 (0%)	0 (0%)	8 (100%)	0 (0%)	8
Provider G	0 (0%)	13 (87%)	0 (0%)	0 (0%)	0 (0%)	2 (13%)	0 (0%)	0 (0%)	15
Total	14 (13%)	18 (16%)	0 (0%)	27 (24%)	6 (5%)	7 (6%)	38 (34%)	2 (2%)	112

Most often received were interventions for nutrition in 80% (90/112) of cases. Support for exercise was received by 76% (85/112) and emotion by 46% (51/112) of cases.

When considering how intervention components were used together, the most frequent combination was all three components, delivered to 34% (38/112), followed by exercise + nutrition to 24% (27/112). The frequency of other combinations of interventions is detailed in Table 7.

There were slight differences in the proportion of cases in aggregated IMD quintiles (IMD 1 and 2 vs. 4 and 5) who received specific prehabilitation components (Table 8). For example, those from the least deprived quintiles were slightly more likely to receive support for exercise and emotion, whilst those from the most deprived quintiles were slightly more likely to receive support for nutrition. However, these differences were not statistically significant (Pearson's *χ* ^2^, all *p* > 0.05). There was a negligible correlation between IMD quintile and the number of prehabilitation components received (Pearson's correlation *r* = 0.05, *p* = 0.61).

**TABLE 8:** Frequency of intervention components received by aggregated IMD quintiles.

Prehabilitation components	Most deprived (IMD 1 and 2) (*n* = 49)	Least deprived (IMD 4 and 5) (*n* = 41)	Pearson *χ* ^2^
Exercise	35/49 (71%)	33/41 (80%)	*p* = 0.319
Nutrition	42/49 (86%)	33/41 (80%)	*p* = 0.508
Emotion	19/49 (39%)	20/41 (49%)	*p* = 0.358

Details of the specific interventions received under each prehabilitation component are presented in Table 9.

**TABLE 9:** Details of the specific prehabilitation components received (total and by each service provider).

Provider	B	C	D	E	F	G	Total
Exercise interventions							
Group exercise (referred or attended)	0 (0%)	12 (34%)	8 (47%)	1 (11%)	0 (0%)	0 (0%)	21 (23%)
Inspiratory Muscle Training	0 (0%)	5 (14%)	0 (0%)	6 (67%)	8 (100%)	0 (0%)	19 (21%)
General exercise advice (non‐individualised)	15 (71%)	3 (9%)	0 (0%)	0 (0%)	0 (0%)	0 (0%)	18 (20%)
Home exercise programme	2 (10%)	9 (26%)	5 (29%)	2 (22%)	0 (0%)	0 (0%)	18 (20%)
1:1 supervised exercise	0 (0%)	0 (0%)	2 (12%)	0 (0%)	0 (0%)	0 (0%)	2 (2%)
Referral to national exercise programme	0 (0%)	1 (3%)	1 (6%)	0 (0%)	0 (0%)	0 (0%)	2 (2%)
Mindful movement/Tai Chi	2 (10%)	0 (0%)	0 (0%)	0 (0%)	0 (0%)	0 (0%)	2 (2%)
Physiotherapist review of exercise/activity	0 (0%)	0 (0%)	1 (6%)	0 (0%)	0 (0%)	0 (0%)	1 (1%)
Patient unwell, e.g., pain/frailty	1 (5%)	3 (9%)	0 (0%)	0 (0%)	0 (0%)	0 (0%)	4 (4%)
Offered exercise intervention but declined	1 (5%)	1 (3%)	0 (0%)	0 (0%)	0 (0%)	0 (0%)	2 (2%)
No intervention required—patient already active	0 (0%)	1 (3%)	0 (0%)	0 (0%)	0 (0%)	0 (0%)	1 (1%)
Total, *n* (denominator for % figures above)	21	35	17	9	8	0	90
Nutrition interventions							
Individualised diet plan or diet review	1 (6%)	2 (6%)	10 (53%)	0 (0%)	0 (0%)	12 (80%)	25 (28%)
Advice to increase protein intake	1 (6%)	14 (45%)	0 (0%)	0 (0%)	7 (88%)	0 (0%)	22 (24%)
General nutritional advice (non‐individualised)	9 (53%)	9 (29%)	0 (0%)	0 (0%)	0 (0%)	0 (0%)	18 (20%)
Advice to increase energy intake	4 (24%)	1 (3%)	0 (0%)	0 (0%)	0 (0%)	1 (7%)	6 (7%)
Oral nutritional supplements	1 (6%)	3 (10%)	0 (0%)	0 (0%)	0 (0%)	1 (7%)	5 (6%)
Managing malnutrition resource issued	0 (0%)	0 (0%)	5 (26%)	0 (0%)	0 (0%)	0 (0%)	5 (6%)
Healthy eating advice	0 (0%)	0 (0%)	1 (5%)	0 (0%)	1 (13%)	0 (0%)	2 (2%)
Dietary advice for symptom management	0 (0%)	1 (3%)	1 (5%)	0 (0%)	0 (0%)	0 (0%)	2 (2%)
Monitoring weight/nutritional status	1 (6%)	0 (0%)	0 (0%)	0 (0%)	0 (0%)	1 (7%)	2 (2%)
Referral to dietitian	0 (0%)	0 (0%)	1 (5%)	0 (0%)	0 (0%)	0 (0%)	1 (1%)
Signposting	0 (0%)	0 (0%)	1 (5%)	0 (0%)	0 (0%)	0 (0%)	1 (1%)
Enteral nutrition	0 (0%)	1 (3%)	0 (0%)	0 (0%)	0 (0%)	0 (0%)	1 (1%)
Total, *n* (denominator for % figures above)	17	31	19	0	8	15	90
Emotion interventions							
General advice (non‐individualised)	12 (60%)	4 (25%)	0 (0%)	1 (100%)	0 (0%)	0 (0%)	17 (30%)
Group session	0 (0%)	5 (31%)	0 (0%)	0 (0%)	6 (75%)	0 (0%)	11 (19%)
1:1 with occupational therapist	0 (0%)	0 (0%)	7 (70%)	0 (0%)	1 (13%)	0 (0%)	8 (14%)
Offered emotional intervention but declined	1 (5%)	5 (31%)	2 (20%)	0 (0%)	0 (0%)	0 (0%)	8 (14%)
Relaxation technique and anxiety management	6 (30%)	0 (0%)	0 (0%)	0 (0%)	0 (0%)	0 (0%)	6 (11%)
Tailored education/information/support	0 (0%)	0 (0%)	1 (10%)	0 (0%)	0 (0%)	2 (100%)	3 (5%)
Fatigue management	0 (0%)	2 (13%)	0 (0%)	0 (0%)	0 (0%)	0 (0%)	2 (4%)
Involvement of family	0 (0%)	0 (0%)	0 (0%)	0 (0%)	1 (13%)	0 (0%)	1 (2%)
Finance support/social services	1 (5%)	0 (0%)	0 (0%)	0 (0%)	0 (0%)	0 (0%)	1 (2%)
Total, *n* (denominator for % figures above)	20	16	10	1	8	2	57

*Note:* Please note that patients may have received more than one intervention so *n* varies.

Abbreviation: *n* = number of interventions.

##### 3.6.2.1 Exercise Interventions

The type of exercise intervention delivered differed by provider, with the online (virtual) or telephone service (Provider B) most often delivering general advice (71% of interventions) and the face‐to‐face multidisciplinary services (Providers C and D) commonly delivering group exercise (34% and 47% respectively) or a home exercise programme (26% and 29%).

##### 3.6.2.2 Nutrition Interventions

The type of nutrition intervention administered also differed by provider. As with exercise, the online (virtual) or telephone service (Provider B) most often delivered general advice on diet (53% of interventions). Two services commonly advised a high protein diet, one dedicated multidisciplinary team (Provider C, 45%) and one service for high risk surgical patients (Provider F, 88%). However, it should be noted that such advice is likely to have also featured in individualised diet plans and advice for increasing nutritional intake. Two services commonly delivered an individualised diet plan or diet review (Provider D, 53% and Provider G, 80%).

##### 3.6.2.3 Emotion Interventions

Similarly, the type of emotional support differed by provider. Support for emotional well‐being was delivered to some patients attending each service, but never in isolation from other prehabilitation components. This was the type of support most often received by the online (virtual) or telephone service (Provider B), when general wellbeing advice was most often given (60% of interventions).

##### 3.6.2.4 Components Received and Cancer Site (Supporting Informationary Material 2)

People with CRC were much more likely to receive ≥ 2 components (82% of patients), as opposed to those with LC (56%) or UGIC (48%). The majority of people with UGIC received a single component (52%), as opposed to LC (44%) and CRC (14%). The most frequent combination for each cancer type was nutrition alone (45%) for UGIC; exercise + nutrition + emotional (41%) for CRC and exercise alone (44%) for LC. There was always an exercise component for those with LC.

## 4 Discussion

The key findings from this medical record review are as follows, and will be further discussed and evidenced in this section. First, a minority of people awaiting cancer treatment access cancer prehabilitation in Wales. Second, the sample of people receiving prehabilitation was unrepresentative of those newly diagnosed with cancer awaiting treatment, with underrepresentation of people from lower socioeconomic groups. Third, the duration, composition and delivery mode of prehabilitation differs by service provider and by cancer site. Finally, access for those from lower socioeconomic groups may have been facilitated in some services, particularly, those that were multidisciplinary, face‐to‐face and more inclusive of different types of cancer.

It was estimated in the current medical record review that 14% of patients accessed prehabilitation services in Wales. In terms of age and gender, the sample was largely typical of the wider UK population with included cancers [25], although women with LC were overrepresented in our sample. Data from a UK‐wide report found that < 3% of patients underwent prehabilitation [26] and a worldwide survey found only 21.1% of hospitals reported prehabilitation to be standard care for all patients undergoing cancer surgery [15]. Prehabilitation is therefore offered to a minority of people with cancer.

However, the proportions observed across IMD (42% in the two most deprived quintiles relative to 38% in the two least deprived quintiles) were not expected, as cancer is most often diagnosed in people from lower socioeconomic groupings [19]. For example, LC incidence is 270% higher in the most deprived compared to the least deprived areas in Wales [16] and CRC is 13% higher [27]. For LC in England, 58% of cases in men and 61% of cases in women were from the most deprived two quintiles [28]. People from areas of socioeconomic deprivation were under‐represented in our study sample. There are a range of possible explanations for this, including lower rates of referral or difficulty accessing prehabilitation.

Although not all providers recorded ethnicity, there was evidence of a lack of ethnic diversity, with all participants who had this item recorded as ‘White’ (Welsh, English, British, Scottish or Northern Irish). This is significant, given that the population living in Wales in 2021 included 2.9% identifying as ‘Asian, Asian Welsh or Asian British’, 1.6% as ‘Mixed or multiple ethnic groups’; 0.9% as ‘Black, Black Welsh, Black British, Caribbean or African’ and a further 0.9% as ‘Other ethnic group’ [29]. Although the incidence of all cancers is highest for ‘White’ ethnicity (533/100 000), this is followed closely by ‘Mixed/multiple ethnic groups’ (513/100 000) and then ‘Other ethnic group’ and ‘Black/African/Caribbean/Black British’ (both 362/100 000) and ‘Asian/Asian British’ (333/100 000) [17, 18]. The lack of evident ethnic diversity in the sample is therefore unexpected. However, it should be noted that 35% of data extraction forms had no ethnicity recorded and it is therefore possible that ethnic diversity was present but simply not recorded.

It was evident that the IMD profile was different across different providers. It is not possible to determine whether this reflects socioeconomic differences in the local populations or whether the referral criteria (including cancer site) or mode of delivery of specific prehabilitation services influences uptake in different socioeconomic groups. For example, it was interesting to observe that the face‐to‐face multidisciplinary services (Providers C and D) and the specialist tertiary service (Provider G) saw the highest proportion of patients from the most deprived groups. People from the most deprived groups were less likely to access the online service (Provider B) or services that were targeted towards specific types of cancer (Providers A, E and F). However, there were multiple points of difference between services and it is therefore difficult to identify specific moderating factors. The impacts of such service design choices need to be more firmly understood if services are to be more inclusive of all people with cancer. It was interesting that IMD did not seem to strongly influence the components of prehabilitation received (Table 7), which might suggest that IMD has a greater influence on access to services than what is received once they are within those services.

The literature would suggest that people from lower socioeconomic groupings have proportionally more comorbidities than those in higher socioeconomic groupings [30]. Comorbidity might present challenges to access and engagement (e.g., duration) in prehabilitation and thus be an explanation for the observed under‐representation of lower socioeconomic groups in the sample. This observation could also reflect the fact that our sample only included patients awaiting treatment (i.e., patients receiving supportive or end of life care who might be expected to have high comorbidity were excluded). Such explanations would also explain the lack of relationship between IMD and CCI in this study sample.

Prehabilitation was found to be characterised by difference across the eight NHS services providing cancer services. There were broadly five different models of prehabilitation including: (1) commission a prehabilitation service from another provider (excluded from this medical note review); (2) target a subgroup of cancer patients at high risk who were therefore expected to benefit from prehabilitation; (3) screen and, if appropriate, refer to a non‐specialist therapy service, for example, community dietitian; (4) offer an online (virtual) or telephone cancer dedicated multidisciplinary prehabilitation service or (5) offer a face‐to‐face dedicated multidisciplinary cancer prehabilitation service. There was also a spectrum of provision, with universal prehabilitation at one end and specifically targeted or specialist prehabilitation interventions at the other, often informed by screening. This variability in prehabilitation service delivery models is also seen globally [15].

The specific components of prehabilitation received differed by service provider and cancer site, as did the duration of prehabilitation. For example, a longer duration was given by services offering dedicated multidisciplinary cancer prehabilitation and people with LC spent longer in prehabilitation compared to people with UGIC or CRC. Duration of prehabilitation will naturally be influenced by the time available before treatment starts [1, 31], but the reason for referral was another influence identified in the current medical review. So, there is a complex interplay of factors impacting prehabilitation duration. We need to better understand the relative importance of prehabilitation components and how they are delivered to optimise outcomes. It is unlikely that one size fits all and prehabilitation should be tailored to individual circumstances [1]. However, there are also likely to be core principles that should underpin all prehabilitation efforts. Different resource constraints and priorities within each organisation will have contributed to the observed variation in service delivery and therefore, homogeneity of the prehabilitation offer is also unlikely. Indeed, a comprehensive international survey of prehabilitation for cancer surgery [15] found that lack of institutional funding and resources were the main reasons for prehabilitation not being offered.

### 4.1 Strengths and Limitations

The medical record review included seven cancer prehabilitation services right across Wales. The services collectively provided cancer prehabilitation services to all but the most rural, where patients were referred to services in England or elsewhere in Wales. Prehabilitation was variably offered to people with the four most common cancer sites (BC, CRC, LC and PC) and another that has been shown to respond well to prehabilitation (UGIC). There were very small numbers of BC and PC patients, perhaps due to established and rapid treatment pathways for these types of cancer. An important limitation is that, despite training and support from the research team, there were differences in the level of detail recorded within the data extraction forms, reflecting the detail documented in individuals' medical records. The variation may have influenced the findings, such as under‐representing the complexity of the prehabilitation offer within some services. This study was unable to determine the reasons for individual patients declining or failing to attend cancer prehabilitation and future research should attempt to elicit such information. Future research should also include outcome measures to inform appropriate health economic evaluation of prehabilitation services. It should be acknowledged that this medical record review only included selected cancer types and services claiming to provide prehabilitation. Individual cancer care workers and wider cancer services in Wales may provide prehabilitation advice and support that has not been captured and services may differ for other cancers. The inherent bias in choosing to only study patients attending prehabilitation means that there is no comparator group, such as patients who were offered prehabilitation and chose not to or were unable to attend or those who never received a prehabilitation offer. The observed heterogeneity in service provision further complicates interpretation of the findings in relation to influences on equity of access, acceptance and adherence. This context is important when attempting to generalise the findings.

## 5 Conclusion

It is evident from this medical note review that only a small proportion (approximately 14%) of patients receive cancer prehabilitation in Wales. Those accessing prehabilitation services were not representative of the population of people in Wales that develop cancer or of the wider population. In particular, people from lower socioeconomic groups were likely to have been under‐represented. Ethnicity was only recorded for 65% and all of these people were recorded as ‘White’. The findings demonstrate a highly variable prehabilitation offer in Wales, with only 34% of patients receiving support for all three components (exercise, nutrition and emotion). So, there are differences in the prehabilitation service offer, which may create health inequity by failing to meet the needs of people at greatest risk of poor outcomes and thus those most likely to benefit from cancer prehabilitation. Further research is required to understand variation in prehabilitation services and how to ensure that prehabilitation is as accessible as possible to all people with cancer. This will enable the targeted efforts required to improve uptake and to enhance cancer treatment outcomes.

## Author Contributions

**Shea Palmer:** writing – original draft, validation, writing – review and editing, formal analysis, project administration, supervision, data curation. **Jane Hopkinson:** conceptualization, investigation, funding acquisition, writing – original draft, methodology, validation, writing – review and editing, formal analysis, project administration, data curation, supervision, resources. **Nichola Gale:** conceptualization, investigation, funding acquisition, writing – review and editing, methodology, supervision. **Nicholas Courtier:** writing – review and editing, funding acquisition, conceptualization, methodology. **Sian Lewis:** conceptualization, funding acquisition, writing – review and editing. **Alexandra Mitchell:** writing – review and editing, validation, methodology, formal analysis, project administration, supervision, investigation, data curation. **Akhilesh Ramachandran:** investigation, methodology, validation, writing – review and editing, formal analysis, data curation. **Manasi Patil:** investigation, writing – review and editing, methodology, project administration.

## Funding

This work was supported by the National Institute for Health and Care Research (NIHR151668).

## Conflicts of Interest

The authors declare no conflicts of interest.

## Supporting information


**Data S1:** Supporting Information Material 1—Data extraction form.
**Supporting Information:** 2—Prehabilitation intervention components delivered by cancer site.

## Data Availability

The data that support the findings of this study are available from the corresponding author upon reasonable request.
