# Supplementary material for: Understanding Equity in Cancer Prehabilitation Services in Wales: A Medical Record Review
Source: Cancer Rep (Hoboken). 2026 Jun 11;9(6):e70598. doi: 10.1002/cnr2.70598 (PMC13255152; doi:10.1002/cnr2.70598)
Supplement: Supplementary file 1 — Data S1: Supporting Information Material 1—Data extraction form. Supporting Information: 2—Prehabilitation intervention components delivered by cancer site. [file CNR2-9-e70598-s001.docx]

**Online Supplementary Material 1 – Data extraction form**

**Data Extraction Form: Patient medical records**

**(All patients attending one clinic for prehab over a four-week period)**

| **Reviewer Details** | | **Coding (researcher)** |
| --- | --- | --- |
| Reviewer name | |  |
| **Record Details** | |  |
| Study reference number (unique patient study ID linked to medical record number) | |  |
| Date of referral for prehab | |  |
| Reason for prehab referral | |  |
| **Diagnosis and treatment** | |  |
| Date of primary diagnosis:  Site of primary diagnosis:  Cancer stage (TNM) at time of primary diagnosis:  Date of diagnosis of metastases (if applicable):  Site of metastases (if applicable):  Treatment for:  □ Primary cancer OR □ Metastases (if applicable)  First line treatment (including adjuvant or neoadjuvant therapy; planned and/or completed – date)  Second line treatment (including adjuvant or neoadjuvant therapy; planned and/or completed – date)  Third line treatment (including adjuvant or neoadjuvant therapy; planned and/or completed – date)  Other treatment (including adjuvant or neoadjuvant therapy; planned and/or completed – date) | |  |
| **Prehab interventions** | |  |
| Management (please list screening/assessment measures with patient score, and treatment and supportive care interventions offered).  First prehab assessment:   \|  \| Assessments \| Interventions \| \| --- \| --- \| --- \| \| Physical activity/exercise/capacity \|  \|  \| \| Diet/nutrition \|  \|  \| \| Emotional support \|  \|  \| \| Other \|  \|  \|   Second prehab consultation:   \|  \| Assessments \| Interventions \| \| --- \| --- \| --- \| \| Physical activity/exercise/capacity \|  \|  \| \| Diet/nutrition \|  \|  \| \| Emotional support \|  \|  \| \| Other \|  \|  \|   Third prehab consultation:   \|  \| Assessments \| Interventions \| \| --- \| --- \| --- \| \| Physical activity/exercise/capacity \|  \|  \| \| Diet/nutrition \|  \|  \| \| Emotional support \|  \|  \| \| Other \|  \|  \|   Fourth prehab consultation:   \|  \| Assessments \| Interventions \| \| --- \| --- \| --- \| \| Physical activity/exercise/capacity \|  \|  \| \| Diet/nutrition \|  \|  \| \| Emotional support \|  \|  \| \| Other \|  \|  \| | |  |
| **Other conditions** | |  |
| Comorbidities (please select)  Myocardial infarction (1)  Congestive heart failure (1)  Peripheral vascular disease (1)  Cerebrovascular disease (1)  Dementia (1)  Chronic pulmonary disease (1)  Connective tissue disease (1)  Peptic ulcer disease (1)  Mild liver disease (1)  Diabetes (1)  Hemiplegia (2)  Moderate or severe renal disease (2)  Diabetes with end organ damage (2)  Any tumour without metastasis (2)  Leukaemia (2)  Lymphoma (2)  Moderate or severe liver disease (3)  Metastatic solid tumour (6)  AIDS (6) | (Weighting for Charlson Comorbidity Index) |  |
| **Outcome** | |  |
| Duration of prehab attendance (time between first and last attendance)  Ongoing: YES/NO  If NO, reason for discontinuation (please select)  Patient choice  Discharge from service  Died  Other | |  |
| **Demographics** | |  |
| Date of birth | |  |
| Sex (please select): Male / Female / Other – please state | |  |
| Postcode (to assess index of multiple deprivation) | |  |
| Ethnicity (please select)  Asian or Asian British   - Indian - Pakistani - Bangladeshi - Chinese - Any other Asian background   Black, Black British, Caribbean or African   - Caribbean - African - Any other Black, Black British, or Caribbean background   Mixed or multiple ethnic groups   - White and Black Caribbean - White and Black African - White and Asian - Any other Mixed or multiple ethnic background   White   - Welsh, English, Scottish, Northern Irish or British - Irish - Gypsy or Irish Traveller - Roma - Any other White background   Other ethnic group   - Arab - Any other ethnic group | |  |
| Main source of income | |  |
| Employment | |  |
| **Other** | |  |
| Comments (if any) | |  |

**Online Supplementary Material 2 - Prehabilitation intervention components delivered by cancer site**

| **Number of intervention components delivered** | | |
| --- | --- | --- |
| 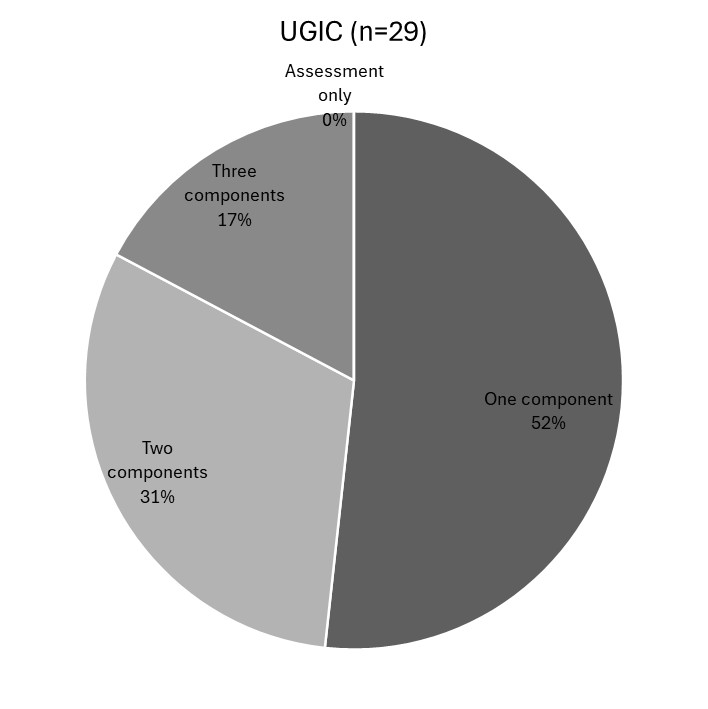 | 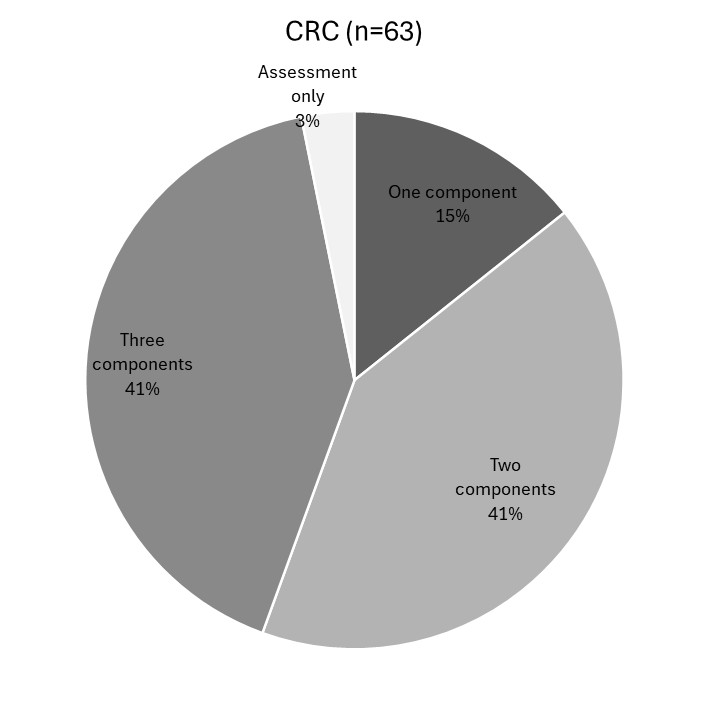 | 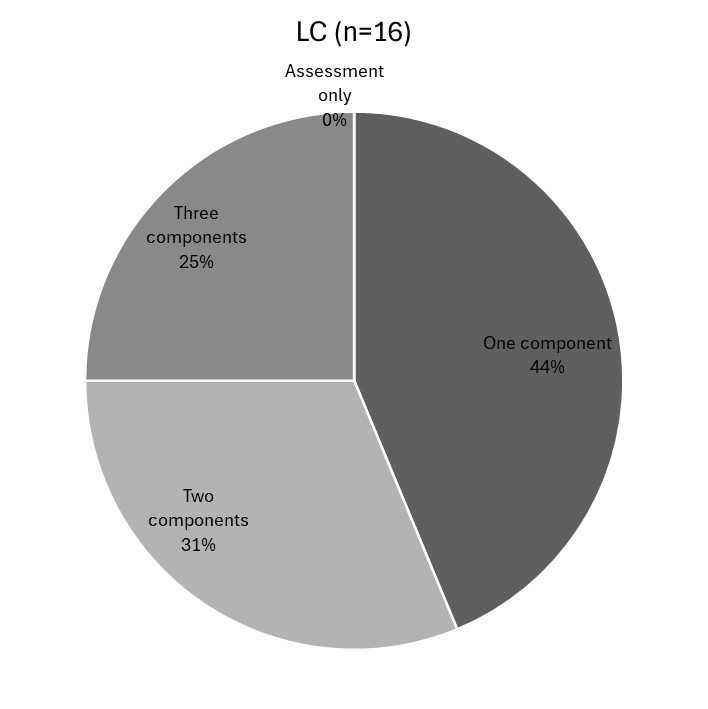 |
| **Combination of intervention components delivered** | | |
| 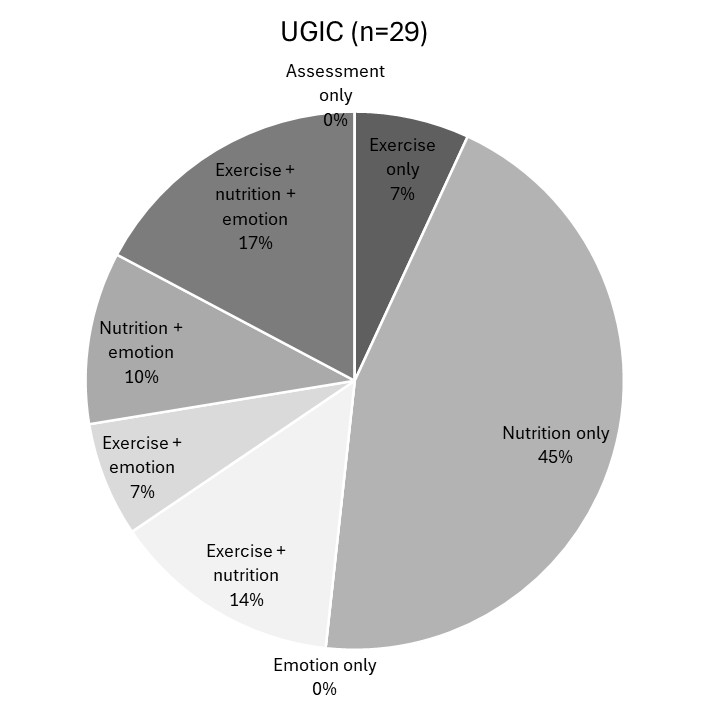 | 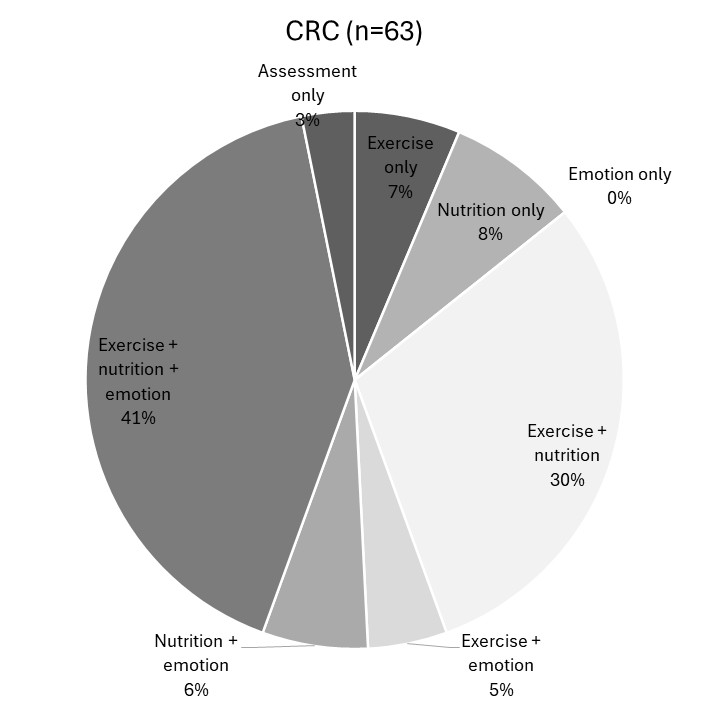 | 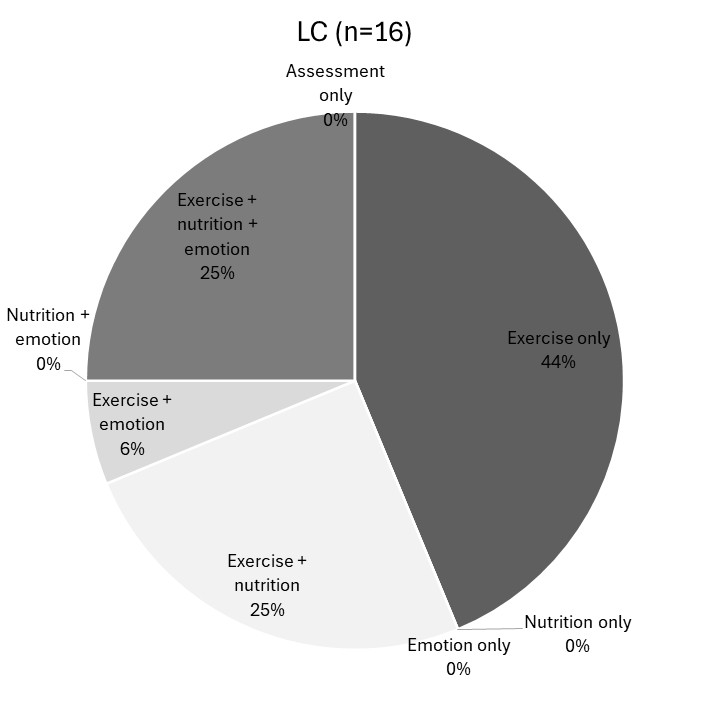 |
